# Current insights in molecular characterization of non-alcoholic fatty liver disease and treatment

**DOI:** 10.3389/fendo.2022.1002916

**Published:** 2022-11-29

**Authors:** Wensheng Che, Ming Zhao, Xiaoqing Li, Chunlong Li, William C. Cho, Shan Yu

**Affiliations:** ^1^ Department of General Surgery, The Second Affiliated Hospital of Harbin Medical University, Harbin, China; ^2^ Chengdu Medical College, Chengdu, China; ^3^ Department of Gastroenterology, The First Affiliated Hospital of Chengdu Medical College, Chengdu, China; ^4^ Department of Pathology, The Second Affiliated Hospital of Harbin Medical University, Harbin, China; ^5^ Department of Clinical Oncology, Queen Elizabeth Hospital, Kowloon, Hong Kong SAR, China

**Keywords:** non-alcoholic fatty liver disease (NAFLD), non-alcoholic steatohepatitis (NASH), gut microbiota, metabolic diseases, adenosine monophosphate-activated protein kinase

## Abstract

There is a continuously rising incidence of non-alcoholic fatty liver disease (NAFLD) around the world, which parallels the increasing incidence of metabolic diseases. NAFLD is a range of liver conditions that contains simple non-alcoholic fatty liver and advanced non-alcoholic steatohepatitis. In serious cases, NAFLD may develop into cirrhosis or even liver cancer. NAFLD has an intense relationship with metabolic syndrome, type 2 diabetes mellitus. It is known that gut microbiota, and functional molecules such as adenosine monophosphate-activated protein kinase JNK, and peroxisome proliferator-activated receptors (PPARs) in progressing and treating NAFLD. Traditionally, the conventional and effective therapeutic strategy is lifestyle intervention. Nowadays, new medicines targeting specific molecules, such as farnesoid X receptor, PPARs, and GLP-1 receptor, have been discovered and shown beneficial effects on patients with NAFLD. In this article, we focus on the molecular mechanisms and therapeutic approaches to NAFLD.

## Introduction

Non-alcoholic fatty liver disease (NAFLD), defined by the National Institute of Diabetes and Digestive and Kidney Diseases, is a condition in which excess fat builds up in the liver, and it is not caused by heavy alcohol consumption. In patients with NAFLD, there is fat accumulation in the liver without inflammation or liver damage. Besides, it is characterized by hepatic steatosis and necroinflammation with different stages of fibrosis known as non-alcoholic steatohepatitis (NASH) (1). The incidence of NAFLD has risen rapidly in the last decade and has become a major health issue: NAFLD affects 25% of the adult population worldwide (2). In addition, NAFLD has seen a significant rise in liver-associated mortality and morbidity among liver-related diseases (3). Notably, NAFLD can occur in non-obese or even lean populations (10.8%) in Asia (4), and NAFLD is associated with extrahepatic diseases, such as cardiovascular disease (CVD) and type 2 diabetes mellitus (T2DM). NAFLD results in insulin resistance due to controlling lipid accumulation and mitochondrial function (5). In advanced stage of NAFLD, NASH is characterized by steatosis, inflammation, and liver damage, often accompanied by pericellular fibrosis (6). NASH elevates the incidence of cirrhosis, hepatic failure, and even hepatocellular carcinoma (HCC). Hyperglycemia and toxic lipids such as ceramides, diacylglycerol (DAG), free fatty acid (FFA), and cholesterol in hepatocytes may result in deleterious effects (glucolipotoxicity), which may change NAFLD from simple steatosis to NASH through mechanisms including cellular death, oxidative stress, and mitochondrial disorders (7).

## Risk factors of non-alcoholic fatty liver disease

### Metabolic diseases

**Figure 1:**
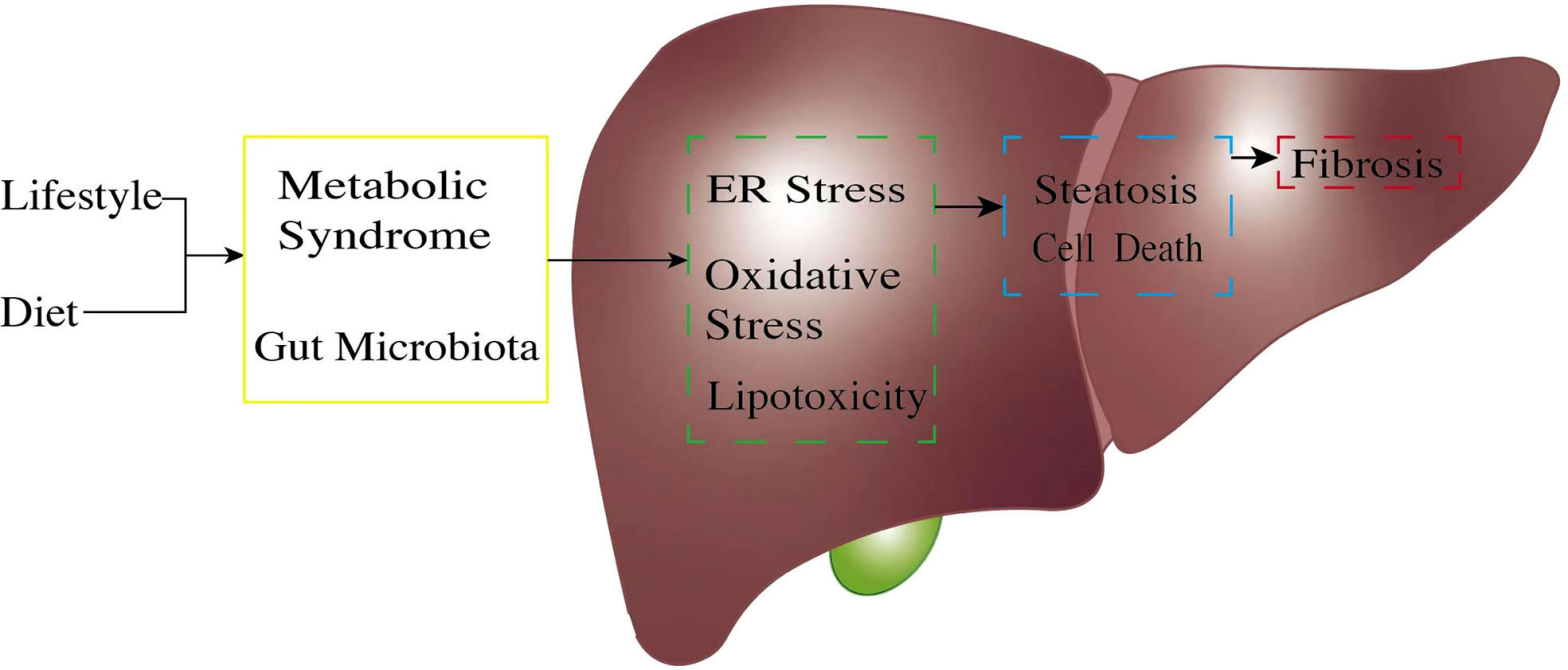
The pathogenic process of non-alcoholic fatty liver disease (NAFLD). The pathogenetic process of NAFLD always starts from unsatisfactory customs in daily life. An unhealthy lifestyle and poor diet, exceptionally high fructose, and a high-fat diet will trigger gut microbiota dysregulation. Unbalanced gut microbiota exposes liver cells to an endotoxic environment by producing short-chain fatty acids and secondary bile acids. At the same time, metabolic syndrome, through factors such as obesity and insulin resistance, causes excessive free fatty acid production because of unsatisfactory energy homeostasis. Once the body reaches its limit for lipid management, this will cause lipid accumulation in the liver cells; expose liver cells to high ER stress, oxidative stress, and lipotoxic condition (green box); and result in hepatocyte inflammation. These pro-inflammatory stimuli generate multiple pathways, leading to further hepatocyte injury (blue box) and continuous inflammation. Cell damage finally causes liver fibrosis (red box). ER, endoplasmic reticulum.

A series of studies have reported that NAFLD is highly associated with metabolic syndrome (8). Metabolic syndrome (hyperglycemia, dyslipidemia, hypertension, and insulin resistance) is a crucial risk factor for NAFLD progression (9, 10). Metabolites of FFAs contribute to hepatocyte injuries such as endoplasmic reticulum (ER) stress, inflammation, apoptosis, and ballooning (11), which finally leads to NAFLD or even NASH (**Figure 1**). Obesity, with reference to body mass index (BMI), has been thought of as a key risk factor for NAFLD for decades. Interestingly, it has been reported that almost 40% of NAFLD patients do not have obesity (12), suggesting that BMI may not be considered a criterion for NAFLD diagnosis. A meta-analysis also showed a much weaker relationship between obesity and the incidence of severe liver diseases compared to other risk factors, such as insulin resistance and dyslipidemia (13). However, the incidence of metabolic syndromes is more prevalent in obese people (14), and NAFLD patients with obesity seem to have a poor prognosis (15). Obesity, or higher BMI, may contribute to NAFLD in a non-directive way, for example, through metabolic syndrome; this requires further in-depth study.

Because of the intense relationship between NAFLD and metabolic diseases, it has been suggested to re-classify fatty liver diseases by whether metabolic dysfunction coexists, naming it as “metabolic dysfunction-associated fatty liver disease (MAFLD)” (16). This classification is probably a better description of patients and improves clinical care and scientific research.

### Gut microbiota

The gut microbiota is an “invisible organ” in the body and a key player in host metabolism and immune regulation (17). The gut microbiota benefits our bodies in several ways, not only contributing to absorbing nutrients and energy, but also producing some molecules, including short-chain fatty acids (SCFAs), secondary bile acids (BAs), and choline derivatives. These molecules can regulate host metabolism through multiple molecular mechanisms (18, 19).

The gut microbiota changes under conditions such as a high-fat diet (HFD), the use of antibiotics, or exposure to toxic substances (20). A recent report also confirmed a strong connection between gut microbiota and NAFLD in mice and humans (21).

NAFLD shows an alteration of the gut microbiota profile, and the occurrence of chronic liver disease is elevated by the overgrowth of extremely small intestinal bacteria (22). For example, the fecal microbiota transplantation (FMP) test demonstrated that the germ-free C57BL/6J mice that transplanted the gut microbiome of HFD mice displayed a higher possibility of steatosis than these transplanted bacteria from normal mice (23).

## Dietary effect for non-alcoholic fatty liver disease

Industrialized nations are bearing witness to an obesity epidemic. An increasing number of patients with serious chronic diseases, such as NAFLD, lead to liver dysfunction and metabolic syndrome, which in turn cause T2DM and vascular complications (24).

In the past decades, the dietary composition in many countries has become focused on higher fat and lower carbohydrates from a traditional diet with a high percentage of carbohydrates and lower fat. The nutritional transition was highly associated with dramatically increased incidence of obesity, NAFLD, T2DM, and cardiovascular disease (25). A high-fat diet could promote NAFLD progression by causing gut microbiota dysregulation (26). A study tested whether a different percentage of fat intake changes the structure of the gut microbiota, decreases the proportion of *Firmicutes*, and increases *Alistipes* and *Bacteroides*. However, a low-fat diet showed contrasting results (27).

### Oxidative stress

Oxidative stress is also strongly associated with NAFLD. Oxidative stress reflects the lack of balance between the reactive oxygen species (ROS) and the eliminating capacity of the antioxidant system (28).

Under physiological conditions, the amount of ROS is kept at a homeostatic status to promote physiological redox signaling. Normal physiological levels of ROS act as signaling molecules involved in cell metabolism, survival, immune defense, proliferation, and differentiation through the modulation of transcription factors and epigenetic pathways (29). However, once under oxidative stress, excessive ROS triggers pathological redox signaling, causing cellular injury in various diseases, significantly when cellular ROS production changes into further toxic ROS species such as HO• (30, 31). Even a little elevation of ROS will lead to cytotoxicity and oxidative stress in the cells. Overproduced ROS can lead to lipid peroxidation, reduced mitochondrial and peroxisomal oxidation of fatty acids, and cytokine release. Furthermore, increased ROS output and oxidative stress are considered underlying mechanisms of insulin resistance (32, 33). Reduction of nuclear factor E2-related factor 2 (Nrf2), a redox-sensitive transcription factor and the principal regulator of the redox balance, and upregulation of the NF-κB signaling pathway have been observed simultaneously with the presence of ROS (32). Also, it has been discovered that the downregulation of Nrf2 and upregulated NF-κB lead to insulin resistance (32). Oxidative stress is associated with insulin resistance, chronic inflammation, and hepatic fibrosis. In addition, it has been shown that the Nrf2 pathway is essential for mitochondrial and ER homeostasis due to its ability to mediate the expression of detoxifying enzymes, which may indicate the relationship between oxidative stress, ER stress, and mitochondrial dysfunction (34). In the condition of NAFLD, impaired redox status and ROS accumulation are the origins of hepatic fat accumulation, thereby leading to hepatic metabolic impairment and NASH progression. Thus, maintaining cellular redox homeostasis is considered one of the therapeutic strategies for NASH (35).

### Inflammation

Inflammation is a process responding to injury or infection, which leads to the secretion of various inflammatory factors, such as cytokines, chemokines, and eicosanoids. Liver inflammatory response is an important factor in NAFLD occurrence and progression. The persistence of inflammatory activity over time results in chronic inflammatory changes that exacerbate tissue injury and result in an abnormal response, which in NAFLD develops into NASH and liver fibrosis (36).

Liver inflammation in NAFLD can be triggered by extrahepatic (e.g., adipose tissue) and/or intrahepatic (e.g., lipotoxicity, oxidative stress, and cell death) factors (36). Studies showed that when under a high-fat diet or upon FFA treatment, Kupffer cells are activated by steatotic hepatocyte released extracellular vesicle and direct effect of FFA, producing a high amount of pro-inflammatory cytokines such as TNF-α and IFN (37). Notably, other cells are also known as important mediators of inflammation and NAFLD progression. NASH is characterized by B-cell and T-cell infiltration of the liver. T cells could potentially stimulate hepatic macrophages regulated through releasing of cytokines, and alterations in regulatory T cell and hepatic dendritic cell homeostasis can trigger immune responses that drive the progression of NASH (38). This evidence supports inflammation being a key pathophysiological mechanism and a target for therapeutic intervention.

### Genetic risk factors

In a family cohort study, the risk of advanced fibrosis in the NAFLD group was 12 times more than in the control group (39). Some single-nucleotide polymorphisms (SNPs), including rs738409 c.444 C>G p.I148, rs58542926 c.499 G>A p.E167K, rs1260326 c.1337 C>T p.P446L, rs641738 g.54173068 C>T/c.50 G>A p.G17E, and rs62305723 c.778 C>T p.P260S, have been discovered as being robustly associated with the pathogenesis of NAFLD (40), and these genetic variants showed moderate-to-large effect sizes in glucose and lipid homeostasis pathways for the development of NAFLD (41). The rs738409 c.444 C>G p.I148 is considered the most common genetic predisposition in NASH. One of the major findings is that the PNPLA3 I148M variant increases susceptibility to the whole spectrum of liver damage related to NAFLD, from steatosis to NASH, fibrosis, and HCC (42). Carrying this variant increases the risk of liver-associated mortality (43). These findings represent that genetic factor is probably an important risk factor for the development of NAFLD. These genes also have an association with therapeutic approaches. For example, PNPLA3 I148M carriers showed higher effectiveness through lifestyle intervention and seemed to receive less benefit from omega-3 supplements than certain other SNPs (44–46).

Recently, targeting these genes to treat NAFLD has come under the spotlight. Targeting PNPLA3 (p.I148M) at RNA levels through small hairpin RNAs (shRNAs) or antisense oligonucleotides (ASOs) could provide long-lasting suppression of the risk variant expression in carriers. PNPLA3 silencing caused a significant reduction of liver steatosis, inflammation, and fibrosis in mice fed with a NASH-inducing diet (47). An ASO compound called ION839 (also known as AZD2693) is currently under investigation in phase I trials (NCT04142424 and NCT04483947).

Degradation of gene production also could be a viable therapeutic intervention. Momelotinib, previously identified to treat myeloproliferative neoplasm, showed downregulation of PNPLA3 mRNA, representing a new and effective therapeutic approach for NASH (48).

The identification of the relationship between genes and the development of NAFLD may explore new therapeutic options, and the future genome-wide studies may reveal additional mechanisms.

## Understanding non-alcoholic fatty liver disease by molecular mechanisms

### Adenosine monophosphate-activated protein kinase

Adenosine monophosphate-activated protein kinase (AMPK) is a well-known energy-sensing kinase that manages the energy balance. Therefore, it is necessary for cell survival with marginal energy supplements (49–51). The AMPK activity is allosterically regulated by AMP/ATP ratio, with activation during nutrition shortage and inactivation under obesity conditions, hyperglycemia (52–54), and hyperinsulinemia (55, 56).

**Figure 2:**
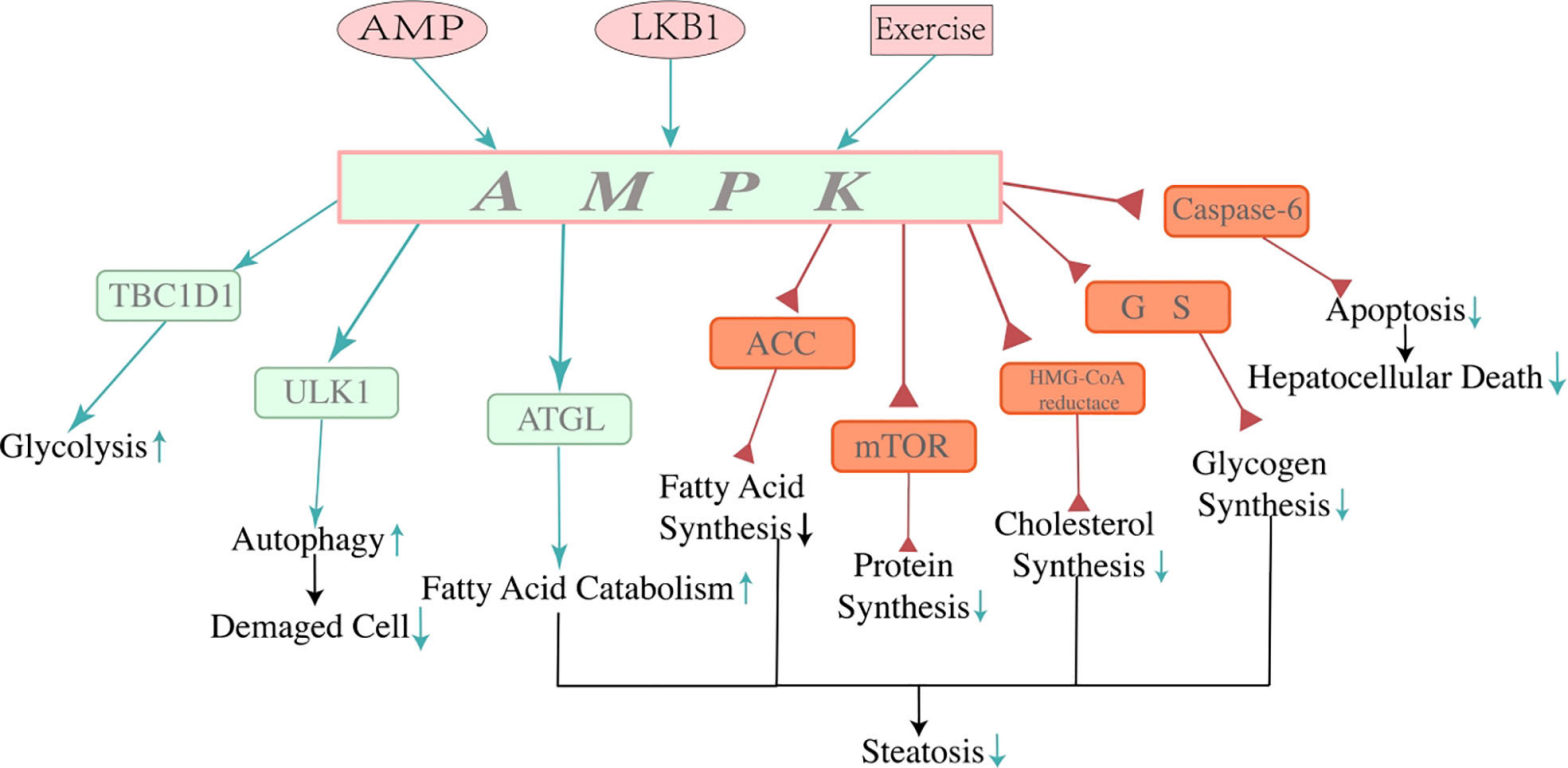
Mechanisms of adenosine 5′-monophosphate-activated protein kinase. AMPK is a crucial controller of energy metabolism. AMP, LKB1, and exercise can activate AMPK directly or indirectly. AMPK regulates protein, fat, and carbohydrate metabolism. Activation of AMPK restrains lipogenesis, promoting fatty acid catabolism in the liver through multiple mechanisms of energy regulation, thus decreasing hepatocyte steatosis and contributing to the consumption of the energy of the whole body. Due to its function in energy regulation, activation of AMPK reduces lipid accumulation by reducing lipid synthesis and stimulating lipid catabolism, which alleviated hepatocyte steatosis and showed a beneficial effect on NAFLD. In addition, the function of AMPK reducing hepatocyte apoptosis by inhibiting activation of caspase-6 has been found recently, which may indicate a new approach to improve NAFLD. Three pink icons at the top of the image are approaches to activating AMPK. The green arrows represent the positive effect, and the red triangles represent the negative effect. NAFLD, non-alcoholic fatty liver disease.

AMPK is activated through phosphorylation on the NH2-terminus (Thr172) (57), which is mediated by its upstream kinase liver kinase B1 (LKB1) or the AMP binding with γ-unit allosterically (58, 59), as well as exercise directly. The AMPK activation not only reduces the AMP/ATP ratio but also induces lipolysis and lipid oxidation to consume whole-body energy (60, 61). Therefore, AMPK activation could benefit NAFLD. AMPK facilitates a variety of biological processes, including oxidative phosphorylation, autophagy, uptake, and utilization of glucose and FFA while reducing anabolism, such as protein synthesis by inhibiting mTOR signaling, cholesterol by inhibiting HMA-CoA reductase, and fatty acids by phosphorylating acetyl CoA carboxylase (ACC) (49, 62–66). These processes are closely correlated with hepatic lipid homeostasis and the pathogenesis of NAFLD. AMPK is dysregulated in obese humans. Decreased AMPK activity in adipose tissue is connected with whole-body insulin resistance, suggesting that AMPK activity in adipose tissue may be necessary for NAFLD (67, 68). In a healthy population, *de novo* lipogenesis (DNL) contributes less than 5% to liver triglyceride (TG) content, while it contributes up to 26% of liver TG in humans with NAFLD (69, 70). Researchers have discovered that AMPK phosphorylates and inactivates ACC to downregulate the rate-limiting step of DNL (50, 71–73). A-769662, an AMPK agonist, showed an abolishment effect in NAFLD, which restored hepatic fatty acid oxidation and ameliorated liver lipid accumulation (74). These studies indicate that AMPK may be a key player in the pathogenesis of NAFLD (**Figure 2**).

The activity of AMPK in hepatocytes is reduced within NAFLD (74). A recent study showed that the downregulation of AMPK in hepatocytes results in more hepatocellular death and fibrosis due to the upregulation of caspase-6 (75). AMPK is phosphorylated by AMPK-related protein kinase 5 (ARK5) and thus prevents hepatocytes from apoptosis by inhibiting the cleavage of procaspase-6 (76, 77). Inversely, when the activity of AMPK is reduced due to overnutrition, hyperglycemia, diabetes, and NAFLD, caspase-6 would gain functions to sustain the caspase cascade. The cascade activates caspase-3 and caspase-7, further leading hepatocytes to caspase-mediated apoptosis (78). Thus, AMPK not only is a crucial energy-sensing protein, but that it may also participate in cell apoptosis regulation in NAFLD/NASH.

### c-Jun N-terminal kinase

c-Jun N-terminal kinase (JNK) is a member of the mitogen-activated protein kinase (MAPK) family and is capable of promoting apoptosis *via* Bcl-2 interacting mediator of cell death (Bim), p53 upregulated modulator of apoptosis (PUMA), and members of pro-apoptotic Bcl-2 family (79). JNK is a vital mediator of insulin resistance and FA-induced lipotoxicity (80), and its activation mediates apoptosis in hepatocytes (81). It has been reported that the ASK-1–JNK axis induced TNF-α related apoptosis in steatotic hepatocytes from a mouse model of NAFLD (56). The prolonged activation of JNK could be alleviated by NF-κB-mediated upregulation of anti-apoptotic genes, such as Bcl-xl and cFLIP (82), which may be a therapeutic strategy for NASH. In conclusion, JNK is essential for regulating apoptosis in NAFLD and NASH. Interestingly, JNK also contributes to protective responses such as hepatocyte proliferation and liver regeneration (83).

### Peroxisome proliferator-activated receptors

**Figure 3:**
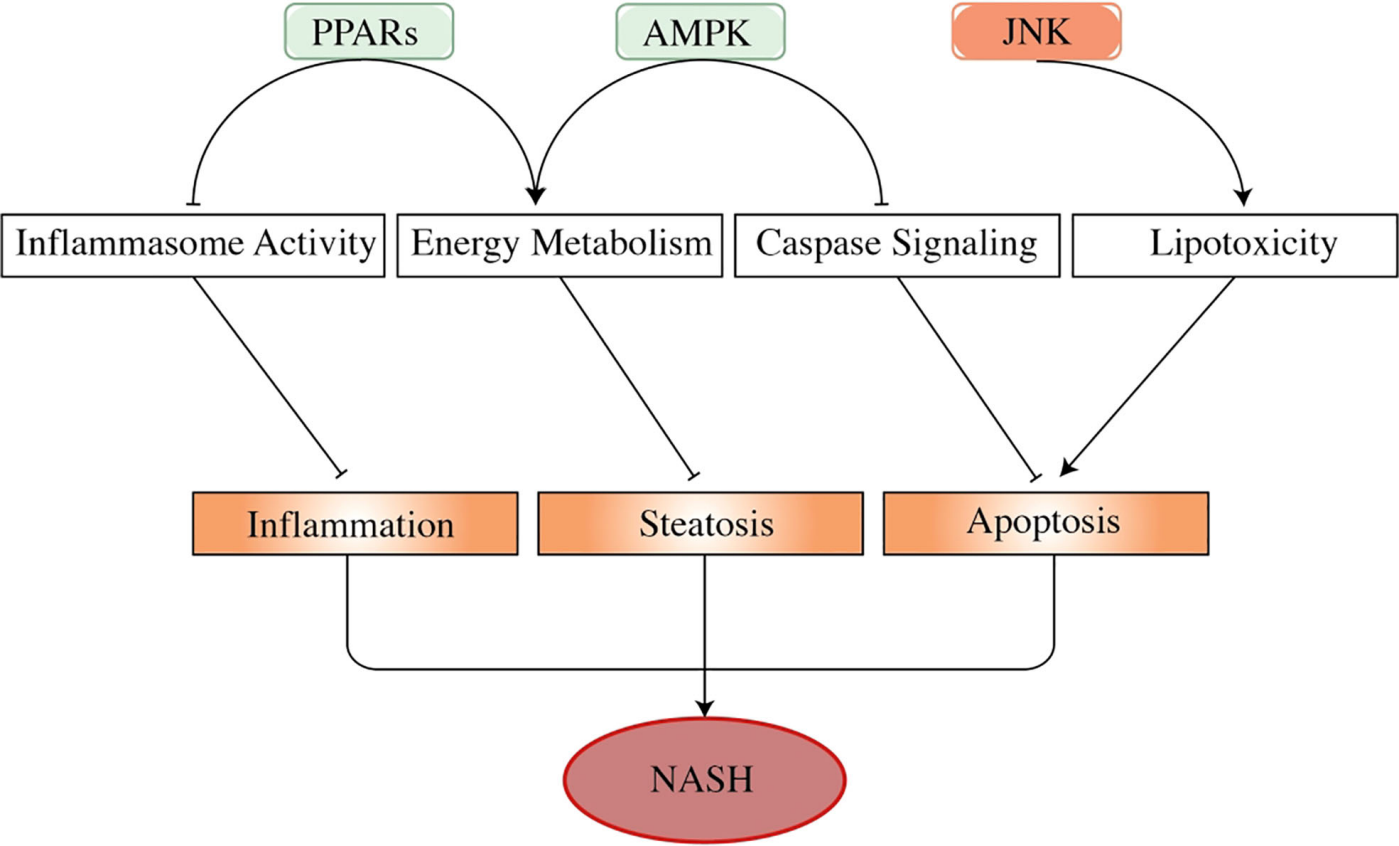
The effects of adenosine 5′-monophosphate-activated protein kinase, peroxisome proliferator-activated receptors, and c-Jun N-terminal kinase in the progress of non-alcoholic steatohepatitis. AMPK, PPARs, and JNK are important factors in NASH progression. As energy sensors, PPARs and AMPK regulate energy usage to prevent the liver from developing steatosis. PPARs also show an inhibitory effect on inflammasome activity (such as TNF-α and IL-6), thus regulating liver inflammation. Activated AMPK attenuates hepatocyte apoptosis by inhibiting the activity of the caspase pathway, especially caspase-6. By contrast, activated JNK promotes liver cell apoptosis by mediating fatty acid-related lipotoxicity and inducing TNF-α activation. These factors play a part in hepatocyte pathogenesis, regulating the progression of NAFLD and NASH. In this figure, arrows represent promoting effect, and short lines imply repressing effect. PPARs, peroxisome proliferator-activated receptors; NASH, non-alcoholic steatohepatitis; NAFLD, non-alcoholic fatty liver disease.

Peroxisome proliferator-activated receptors (PPARs) belong to the nuclear receptor superfamily, which is considered a fatty acid sensor, thus modulating carbohydrate metabolism and energy usage (**Figure 3**). PPARs are also reported as regulators of NAFLD development and treatment (84–86). PPARα, an isoform of PPARs, is negatively correlated with the severity of NASH (87), which may be linked with metabolism modulation, such as fatty acid metabolism, ketogenesis, and β-oxidation (88, 89). In addition, PPARα showed a countering effect on pro-inflammatory cytokines that were characterized by the downregulation of IL-6, TNF-α, and COX2 (90). In the PPARα knock-out mice, it showed an increment in triglyceride, oxidative stress, inflammation, and hepatocyte death (91). The PPARα activation was shown to improve NAFLD through AMPK-PPARα signaling through aerobic training (92). PPARγ is predominantly expressed in adipose tissue and macrophages and is capable of regulating adipocyte differentiation, lipid synthesis, and lipolysis (93). In NAFLD, disrupted PPARγ restricted macrophage polarization to the M2 phenotype, thus inducing hepatocyte steatosis, inflammation, and fibrosis. Thiazolidinediones (TZDs), a PPARγ agonist, were reported to affect NASH by increasing insulin sensitivity (94, 95).

## Therapeutic strategies

Although multiple clinical trials for NAFLD/NASH have been in process in recent years, there is currently no drug that has been approved by the United States Food and Drug Administration (U. S. FDA) (6). The current most effective way to alleviate NAFLD is still exercise and dietary intervention (96). Meanwhile, the drugs already approved for treating other diseases are repurposed for NAFLD/NASH treatment, saving time and cost. Notably, phase III clinical trials of promising drugs of these kinds are ongoing.

### Exercise and dietary intervention

Lifestyle (exercise and dietary) intervention is still a good therapeutic choice for NAFLD. Several studies have demonstrated that caloric restriction and exercise could improve NAFLD through weight loss (97). Lifestyle intervention showed an impressive reduction in alanine aminotransferase (ALT) and aspartate aminotransferase (AST) and amelioration in steatosis and cirrhosis (97). In addition, exercise stimulates AMPK directly, which restricts the synthesis of fatty acid, promotes fatty acid catabolism, and ameliorates hepatocyte apoptosis (75, 98). In addition, lifestyle intervention could reduce inflammation, ballooning, and fibrosis. However, achieving NASH improvement through weight loss has become challenging because of work-related stress and the difficulty of changing lifestyles in the modern era (99).

In addition to caloric restriction, the rational dietary structure is a hotspot for NAFLD/NASH improvement. The rampancy of NAFLD shows a parallel increase in the prevalence of a diet that contains high fat and low fiber. In contrast, a diet that contains whole grains with a high proportion of fiber showed a great effect on weight loss and blood lipid profile (100). It also modulates the composition of gut microbiota (49), which may further have an impact on NAFLD.

It has been reported that ω-3 poly-unsaturated fatty acids (PUFAs) could ameliorate liver fat and AST (101), and a lower content of ω-3 PUFAs was detected in patients with NAFLD (102). These findings highlighted made the diet with higher content of mono-unsaturated fatty acids (MUFAs) and ω-3 fatty acids a hotspot, such as nuts, olive oil, fish, and wine. Compared with other types of diet, these foods showed impressive results in weight loss, reduction of ALT, and improvement of insulin resistance in NAFLD patients (103, 104).

## Medications in advance

### Obeticholic acid

**Figure 4:**
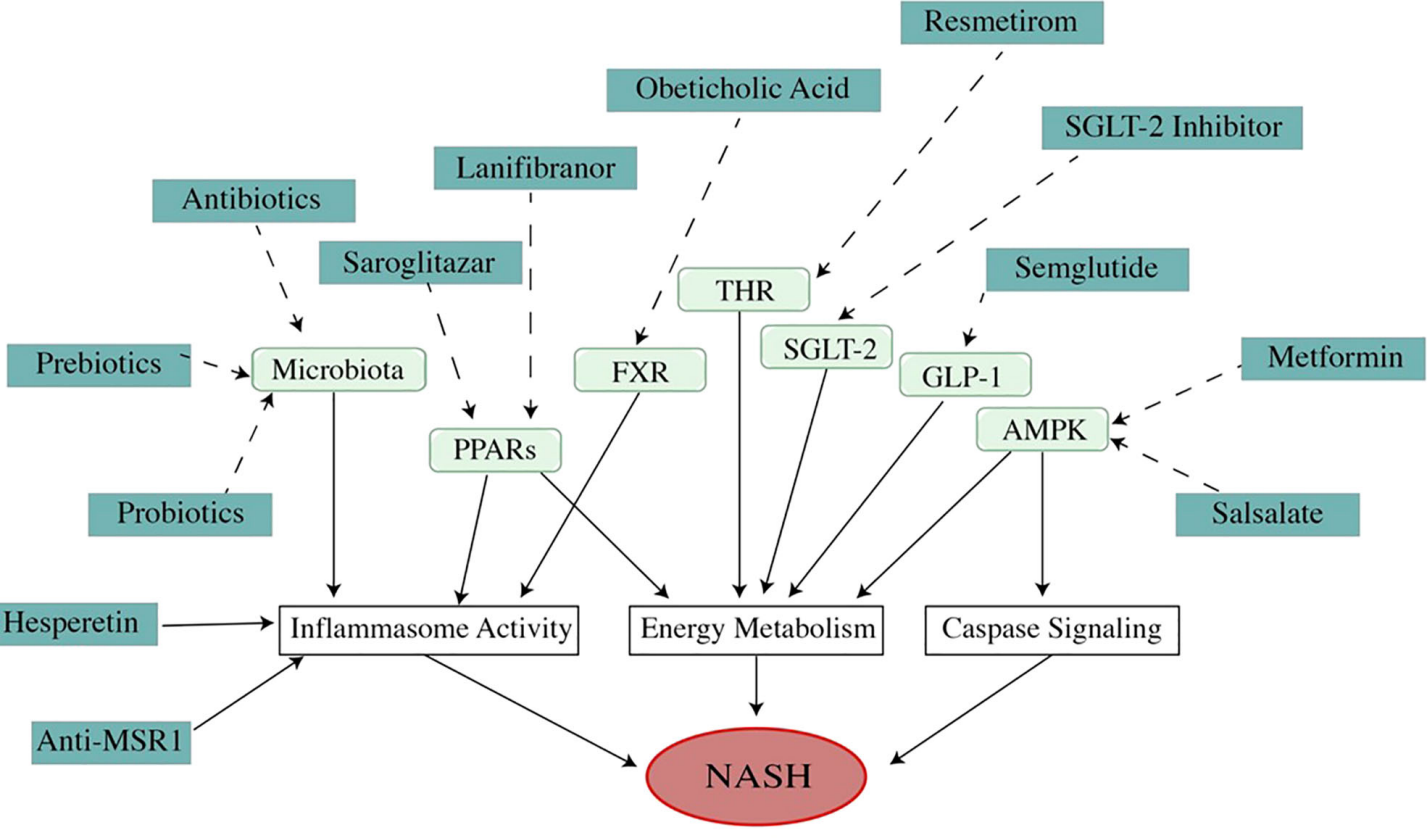
Mechanism of medications (dosage is listed in **Table 1**). As a multi-factor disease, the pathogenesis of NAFLD/NASH involves many pathways. Modulating these pathways with different medications can improve NAFLD/NASH. As a metabolic disease, the theme of NAFLD treatment is to regulate energy metabolism. A high percentage of ongoing drug experiments are related to energy management. However, the medicines that improve NAFLD in other mechanisms, such as regulating apoptosis or inflammation, are also attracting attention because of their impressive effects in treatment. Unfortunately, all of these drugs are undergoing trials, and no drug has been approved by U. S. FDA).

Obeticholic acid is a selective farnesoid X receptor agonist. Activation of the farnesoid X receptor can reduce fibrosis and inflammation in NASH by regulating bile acid metabolism (**Figure 4**) (105, 106). Usage of obeticholic acid for 18 months showed a significant amelioration in fibrosis and histological problems such as hepatocyte ballooning, lobular inflammation, and reduction of ALT and AST level in a phase III trial (NCT02548351) (107). Obeticholic acid showed mild adverse effects, but the most common one is pruritus. Another mechanism of obeticholic acid in NASH treatment has been discovered that inhibits NLRP3 inflammasome in macrophage activation and suppresses lipid accumulation without the participation of the farnesoid X receptor (108).

**Table 1:** Medications treatment.

Medication	Dosage	Outcome and proposed mechanism	Experimental model	Ref. no.
Obetecholic acid	Daily 10mg for 18 months	Regulates bile acid, improves ALT, AST, fibrosis, ballooning, inflammation	Human	105–108
	Daily 25mg for 18 months
Lanifibranor	Daily 800mg for 6 months	Improves fibrosis, inflammation, ALT, AST, apoptosis by activating PPARs	Human	109, 110
	Daily 1200mg for 6 months
Resmetirom	Daily 80mg for 12 weeks	Reduces liver fat, cholesterol by activating THR-β	Human	111, 112
	Daily 89mg for 36 weeks
Semaglutide	Daily 0. 1mg for 72 weeks	Improves ALT, AST, triglycerides, body weight via activating GLP-1	Human	113
	Daily 0. 2mg for 72 weeks			
	Daily 0. 4mg for 72 weeks			
Prebiotics	N/A	Decreases serum endotoxin, ALT, AST, oxidative stress, inflammation by changing gut microbiota	HFD induced NAFLD model Human	114, 115
Probiotics	N/A	Reduces lipid accumulation, oxidative stress, TNF-α, IL-1β		116, 117
Metformin	500mg three times a day for 4 months	Reduces BMI, ALT via restriction of lipid accumulation and stimulating AMPK	Human	118, 119
Salsalate	Daily 300mg/kg for 7days	Reduces fat mass, reverses AMPK repression and caspase-6 activation	C57BL/6J mice	120
Saroglitazar	Daily 1mg for 16 weeks	Improves ALT, insulin resistance, fibrosis, and dyslipidemia by stimulating PPARα/γ	Human	121
	Daily 2mg for 16 weeks			
	Daily 4mg for 16 weeks			
Hesperetin	N/A	Alleviate plasma lipid profile, Reduce hepatic ROS overproduction	HepG2 cell	122
			HDF mice	

ALT, alanine aminotransferase; AST, aspartate aminotransferase; GLP-1, Glucagon-like peptide 1; BMI, Body mass index; TNF-α, tumor necrosis factor alpha; IL-1β, Interleukin-1β; AMPK, adenosine 5‘-monophosphate-activated protein kinase; PPAR, Peroxisome proliferator-activated receptor; ROS, Reactive oxygen species; N/A, Not applicable.

### Lanifibranor

Lanifibranor is an anti-fibrotic drug that activates PPAR-α, PPAR-β, and PPAR-δ, called a pan-PPAR agonist. Lanifibranor improved insulin resistance, fibrosis, and inflammation in preclinical trials (109, 110). Oral intake of 800 or 1,200 mg once daily of lanifibranor for 6 months showed impressive amelioration in steatosis, activity, and fibrosis (SAF) score, ALT, AST level, and markers of apoptosis and steatosis in a phase IIb trial (NCT03008070) (123). Some scholars suggest that there is no sufficient evidence to support the efficacy of lanifibranor because there is only one randomized controlled trial for the drug (124). A phase III trial of lanifibranor in the treatment of NASH is ongoing to investigate its safety and long-term efficacy.

### Resmetirom

Resmetirom is a thyroid hormone receptor β (THR-β) agonist. THR-β is the most important receptor of thyroxine in the liver, regulating cholesterol metabolism (111). Many studies demonstrated that activation of THR-β reduces triglyceride, cholesterol, apoptosis, and insulin resistance in an animal model (125–127). Usage of resmetirom for 12 or 36 weeks orally significantly alleviated liver fat content evaluated by MRI-PDFF in a phase II trial (NCT02912260) (112). At the same time, resmetirom showed relatively mild side effects (diarrhea and nausea). Resmetirom has also been demonstrated to reduce ALT and AST reduction. A phase III trial is ongoing to investigate its efficacy on NASH and stage F2–F3 fibrosis patients.

### Semaglutide

Semaglutide is a glucagon-like protein-1 (GLP-1) receptor agonist approved for T2DM treatment and chronic weight management (128). A number of trials have recently assessed the possible beneficial hepatic effects of injectable GLP-1 receptor agonists for NAFLD. An updated meta-analysis of randomized controlled trials showed that GLP-1 receptor agonists reduce liver fat content and serum liver enzyme level, thus improving NAFLD (129). Semaglutide has shown its therapeutic effect for NASH in phase II clinical trials in 2021 (113). Compared with the placebo, it showed the ability to ameliorate obesity, T2DM, ALT, AST, fibrosis, and liver histology. However, semaglutide also showed side effects, such as constipation, nausea, vomiting, decreased appetite, and abdominal pain. Moreover, neoplasm and fatal cardiovascular events were observed during the trial. The American Association for the Study of Liver Disease practice guidelines published in 2018 suggested that it is premature to consider GLP-1 receptor agonists to treat NAFLD or NASH (130). Nevertheless, at the same time, some researchers suggest that the effect of GLP-1 receptor agonists for NAFLD treatment is attractive, especially in patients with coexisting type 2 diabetes or obesity (124). The usage of semaglutide for NAFLD requires further observation.

### Saroglitazar

Saroglitazar is a PPARα/γ agonist shown to improve NAFLD/NASH (131). Due to its double-sensitizing effect, saroglitazar not only increases β-oxidation and reduces triglyceride synthesis but also increases insulin sensitivity (121). In a randomized controlled, double-blind phase II trial, saroglitazar ameliorated ALT secretion, insulin resistance, and dyslipidemia significantly in NAFLD/NASH patients (132). Of note, even though limited cases of severe adverse effects were observed, a mild, dose-dependent weight gain was observed in the saroglitazar group.

### Metformin

Metformin is widely used in T2DM as a first-line therapy, which suppresses hepatic gluconeogenesis (133–135). Metformin showed an inhibitory effect in lipogenesis and lipid oxidation in hepatocytes (74), restricting lipid accumulation in NAFLD. Metformin showed a significant effect on weight loss in a human trial (136) and an amelioration effect on ALT levels (118, 119). In addition, metformin activates AMPK through the phosphorylation at Thr172 to inhibit gluconeogenesis in the liver (137). Meanwhile, metformin inhibited the cleavage and activation of pro-apoptotic factors, such as caspase-3 and caspase-7, thus ameliorating apoptosis in the NASH model (138). Meanwhile, some guidelines recommend against using metformin as a specific treatment for NASH because metformin was shown to not improve liver histology in adult NAFLD patients (124).

### Salsalate

Salsalate, a member of the salicylates family, is a weak non-steroidal anti-inflammatory drug (NSAID) used for inflammatory and non-inflammatory disorders (139, 140). It has been reported that salsalate strongly phosphorylated AMPK and ACC and restored activation of AMPK and caspase-6 improved NAFLD in the HFD mouse model (120). Additionally, salsalate elevates the resting expenditure of energy (141, 142). These mechanisms indicate that salsalate may improve symptoms of NASH/NAFLD simultaneously. Furthermore, salsalate may also contribute to NASH improvement *via* COX-2, which may interact with TNF-α and IL-6 to promote hepatocellular apoptosis (143).

### Antibiotics

Several types of antibiotics are being tested for NAFLD counteraction due to the association between gut microbiota and fatty liver disease. Long-term usage of antibiotics reduces gut bacteria diversity and downregulates liver inflammation (144). Rifaximin, an antibiotic that is non-absorbable in the intestines, induced a significant reduction of AST, ALT, low-density lipoprotein (LDL), and BMI in NASH patients (145). However, antibiotic application in NAFLD is of concern because antibiotics not only kill the pernicious bacteria, but also destroy the probiotics. Moreover, the most concern issue is antibiotic resistance due to frequent abuse in daily life.

### Prebiotics

Prebiotics are incompletely digested food ingredients that guide gut microbiota in a beneficial manner (146). Prebiotic feeding is an effective therapeutic strategy for NAFLD that works by increasing the population of probiotics in the gut (114, 115). The underlying mechanism may include oxidative stress reduction, inflammation alleviation, glucose tolerance, and triglyceride accumulation by adjusting gut microbiota (147–149). In addition, prebiotics also stimulate SCFA production, which is beneficial for NAFLD.

### Probiotics

Numerous animal studies, as well as clinical trials, have demonstrated the effect of probiotics on NAFLD improvement. Probiotics are a series of non-pathogenic microbes that positively impact the host (116). Probiotics improve NAFLD by reducing lipid accumulation, oxidative stress, and inflammatory cytokines such as TNF-α and IL-1β (116, 117). However, probiotics are raising concerns because of biosafety, and just a few bacteria have been proven to have therapeutic effects. Personalized use of probiotics based on gut microbiota tests may be an effective way of NAFLD treatment because of the different gut microbiota biological structures among individuals.

### Hesperetin

Hesperetin (3′,5,7-trihydroxy-4′-methoxyflavanone) is a citrus flavonoid belonging to the flavanone class and is abundant in oranges, lemons, and grape juice. A recent study discovered that hesperetin alleviated oleic acid-induced hepatotoxicity and oxidative stress *in vitro* and plasma lipid profile, including TG, total cholesterol (TC), and LDL-C, in HFD-induced NAFLD rat model (122). Hesperetin showed its effect in reducing fatty acid-induced hepatic ROS overproduction and oxidative damage. Also, hesperetin showed inhibition of fatty acid-induced NF-κB activation and subsequent inflammation by reducing ROS overproduction (122). Anti-macrophage scavenger receptor-1 (anti-MSR1) antibody (MSR1 inhibitor) may be an important therapeutic approach for the treatment of NAFLD requiring clinical investigations.

### Anti-MSR1 antibody

MSR1 is an important receptor for the uptake of lipids in macrophages, leading to an inflammatory response and metabolic changes throughout the body. An MSR1 antibody showed a reduction of hepatic inflammation and changes in hepatic lipid metabolism by reducing hepatic lipid-laden foamy macrophages *in vivo* and *ex vivo* (150). Targeting MSR1 using monoclonal antibody therapy in an obesity-associated NAFLD mouse model and human liver slices displayed prevention of foamy macrophage formation and inflammation by regulating the JNK signaling pathway (150). MSR1 plays a critical role in lipid-induced inflammation and the MSR1 inhibitor may be an interesting therapeutic approach for the treatment of NAFLD in the future.

### Sodium-glucose cotransporter-2 inhibitors

Sodium-glucose cotransporter-2 (SGLT-2) inhibitors (such as dapagliflozin, empagliflozin, ipragliflozin, and canagliflozin) are oral glucose-lowering medicines approved for the treatment of T2DM. Recent studies showed beneficial effects in people with NAFLD (124). By reducing the renal capacity to reabsorb filtered glucose, SGLT-2 inhibitors lessen the ability to reabsorb filtered glucose in the kidney, thereby lowering serum glucose and accompanying some additional benefits such as weight loss and blood pressure control (151, 152). Recently, a meta-analysis of several placebo-controlled or randomized control trials that used various SGLT-2 inhibitors for the treatment of NAFLD has displayed that usage of SGLT-2 inhibitor for 24 weeks significantly decreased ALT, gamma-glutamyl transferase (GGT) level, and liver fat content, as well as body weight in NAFLD patients (152).

## Conclusion and future perspective

NAFLD has become one of the major public health issues throughout the world. The rampancy of NAFLD worldwide is highly associated with a changed lifestyle and rising incidence of metabolic problems such as obesity or T2DM. We should pay attention to dietary structure and exercise to prevent metabolic disease and NAFLD. Exercise and dietary intervention is the current therapeutic strategy Nevertheless, the pace of newly discovered molecules correlated with NAFLD, such as AMPK, PPARs, and JNK, may act as alternative approaches to NAFLD treatment. Researchers are focusing on the discovery of medications that regulate energy metabolism. In the future, combination therapy with dietary intervention, exercise, and medicine will probably be the mainstream and effective therapeutic strategy because NAFLD is a complex disease that involves many pathways in pathogenesis. Besides, liver biopsy is regarded as the most reliable diagnostic approach. However, a biopsy is a snapshot which involves invasive operation and cannot provide continuous monitoring of changes in disease. Therefore, a repeatable, non-invasive approach that is capable of precisely diagnosing and staging is needed. Some extracellular biomarkers, such as circulating nucleic acid fragments, have been identified that could help NAFLD diagnosis. Cell-free non-coding RNA showed its different expression profile among different stages of NAFLD patients (153). Recent studies have discovered that plasma exosome and some exosome markers such as CD9, CD36, and CD63 are significantly increased in NAFLD patients, especially those liver-derived exosome markers, compared with controls (154, 155). Exosomes are spotlighted as an effective target for NAFLD diagnosis due to their effect on lipid metabolism. These newly discovered biomarkers are showing their potential in recent studies but still need further validation. Also, low-cost pharmacotherapy is required because of the relation to lifestyle and the prevalence of NAFLD. We look for reliable and cost-effective diagnostic approaches because a considerable number of patients worldwide are not properly diagnosed. We summarized our views on potential research directions for NAFLD in **Table 2**.

**Table 2:** Potential future perspectives.

Screening and diagnosis	1. Explore novel non-invasive biomarkers or an index that included several biomarkers to easily and accurately screen and diagnose NAFLD. 2. Optimize or create an image-based AI analysis with accuracy close to that of liver biopsy in the diagnosis of NAFLD and liver fat content as much as possible and is cost-saving. 3. A non-invasive or minimally invasive way to continuously and accurately monitor the liver fat content and treatment response of NAFLD (liquid biopsy, etc.).
Mechanism of the pathogenesis of NAFLD	1. The reason for the excessive fat accumulated in liver. 2. Is there a decisive mechanism in the pathogenesis of NAFLD among the mechanisms we already know? 3. The reason for the increasing number of NAFLD in non-obese population. 4. Is there a key substance controlling fat metabolism (like insulin in glucose metabolism)?
Genetic factor	1. Classify NAFLD genotype by signature gene expression. 2. Design personalized treatment guided by different genotypes. 3. Are there any genes making people susceptible to NAFLD?
Therapy	1. Discover drugs against hepatocyte inflammation and apoptosis by targeting AMPK–caspase-6 axis only in liver. 2. Check the effect of antibiotics on NAFLD further. 3. Are vaccines or other immunological ways possible for preventing NAFLD?

NAFLD, non-alcoholic fatty liver disease.

## Author contributions

WSC drafted the manuscript and figures. MZ conducted the literature research and revised the entire manuscript language. XL revised the manuscript and figures. CL acquired the funding and conducted the concept design. SY and WCC revised the manuscript and supervised the study.

## Funding

Funding was provided by the Postdoctoral Scientific Research Developmental Fund (No. LBH-Q20112) of Heilongjiang.

## Conflict of interest

The authors declare that the research was conducted in the absence of any commercial or financial relationships that could be construed as a potential conflict of interest.

## Publisher’s note

All claims expressed in this article are solely those of the authors and do not necessarily represent those of their affiliated organizations, or those of the publisher, the editors and the reviewers. Any product that may be evaluated in this article, or claim that may be made by its manufacturer, is not guaranteed or endorsed by the publisher.

## Glossary

**Table:**

ACC	acetyl CoA carboxylase
AMP	adenosine monophosphate
ARK5	AMPK-related protein kinase 5
ASO	antisense oligonucleotide
ATP	adenosine-triphosphate
Bas	bile acids; Bcl-2, B-cell lymphoma 2
BH3	Bcl-2 homology 3
BMI	body mass index
cFLIP	Cellular FLICE (FADD-like IL-1β-converting enzyme)-inhibitory protein
COX2	cyclooxygenase-2
CVD	cardiovascular disease
DNL	*de novo* lipogenesis
ER	endoplasmic reticulum
FDA	Food and Drug Administration
FFA	free fatty acid
FMP	fecal microbiota transplantation
GLP-1	glucagon-like protein-1
HCC	hepatocellular carcinoma
HFD	high-fat diet
HMA-CoA	β-hydroxy β-methylglutaryl-CoA
IL-6	interleukin-6
IR	insulin resistance
JNK	c-Jun N-terminal kinase
LDL	low-density lipoprotein
LKB1	liver kinase B1
MAFLD	metabolic dysfunction-associated fatty liver disease
MAPK	mitogen-activated protein kinase
MUFAs	mono-unsaturated fatty acids
MSR1	macrophage scavenger receptor-1
NAFLD	non-alcoholic fatty liver disease
NASH	non-alcoholic steatohepatitis
NF-κB	nuclear factor κB
Nrf2	nuclear factor E2-related factor 2
NSAIDs	non-steroidal anti-inflammatory drugs
PPARs	peroxisome proliferator-activated receptors
PUMA	p53-upregulated modulator of apoptosis
ROS	reactive oxygen species
SCFAs	short-chain fatty acids
SNP	single-nucleotide polymorphism
shRNA	small hairpin RNA
SLGT-2	sodium-glucose cotransporter-2
T2DM	type 2 diabetes mellitus
TG	triglyceride
TNF-α	tumor necrosis factor-α
TZDs	thiazolidinediones
VLDL	very low-density lipoprotein
ACC	acetyl CoA carboxylase
AMP	adenosine monophosphate
ARK5	AMPK-related protein kinase 5
ASO	antisense oligonucleotide
ATP	adenosine-triphosphate
Bas	bile acids; Bcl-2, B-cell lymphoma 2
BH3	Bcl-2 homology 3
BMI	body mass index
cFLIP	Cellular FLICE (FADD-like IL-1β-converting enzyme)-inhibitory protein
COX2	cyclooxygenase-2
CVD	cardiovascular disease
DNL	*de novo* lipogenesis
ER	endoplasmic reticulum
FDA	Food and Drug Administration
FFA	free fatty acid
FMP	fecal microbiota transplantation
GLP-1	glucagon-like protein-1
HCC	hepatocellular carcinoma
HFD	high-fat diet
HMA-CoA	β-hydroxy β-methylglutaryl-CoA
IL-6	interleukin-6
IR	insulin resistance
JNK	c-Jun N-terminal kinase
LDL	low-density lipoprotein
LKB1	liver kinase B1
MAFLD	metabolic dysfunction-associated fatty liver disease
MAPK	mitogen-activated protein kinase
MUFAs	mono-unsaturated fatty acids
MSR1	macrophage scavenger receptor-1
NAFLD	non-alcoholic fatty liver disease
NASH	non-alcoholic steatohepatitis
NF-κB	nuclear factor κB
Nrf2	nuclear factor E2-related factor 2
NSAIDs	non-steroidal anti-inflammatory drugs
PPARs	peroxisome proliferator-activated receptors
PUMA	p53-upregulated modulator of apoptosis
ROS	reactive oxygen species
SCFAs	short-chain fatty acids
SNP	single-nucleotide polymorphism
shRNA	small hairpin RNA
SLGT-2	sodium-glucose cotransporter-2
T2DM	type 2 diabetes mellitus
TG	triglyceride
TNF-α	tumor necrosis factor-α
TZDs	thiazolidinediones
VLDL	very low-density lipoprotein
ACC	acetyl CoA carboxylase
AMP	adenosine monophosphate
ARK5	AMPK-related protein kinase 5
ASO	antisense oligonucleotide
ATP	adenosine-triphosphate
Bas	bile acids; Bcl-2, B-cell lymphoma 2
BH3	Bcl-2 homology 3
BMI	body mass index
cFLIP	Cellular FLICE (FADD-like IL-1β-converting enzyme)-inhibitory protein
COX2	cyclooxygenase-2
CVD	cardiovascular disease
DNL	*de novo* lipogenesis
ER	endoplasmic reticulum
FDA	Food and Drug Administration
FFA	free fatty acid
FMP	fecal microbiota transplantation
GLP-1	glucagon-like protein-1
HCC	hepatocellular carcinoma
HFD	high-fat diet
HMA-CoA	β-hydroxy β-methylglutaryl-CoA
IL-6	interleukin-6
IR	insulin resistance
JNK	c-Jun N-terminal kinase
LDL	low-density lipoprotein
LKB1	liver kinase B1
MAFLD	metabolic dysfunction-associated fatty liver disease
MAPK	mitogen-activated protein kinase
MUFAs	mono-unsaturated fatty acids
MSR1	macrophage scavenger receptor-1
NAFLD	non-alcoholic fatty liver disease
NASH	non-alcoholic steatohepatitis
NF-κB	nuclear factor κB
Nrf2	nuclear factor E2-related factor 2
NSAIDs	non-steroidal anti-inflammatory drugs
PPARs	peroxisome proliferator-activated receptors
PUMA	p53-upregulated modulator of apoptosis
ROS	reactive oxygen species
SCFAs	short-chain fatty acids
SNP	single-nucleotide polymorphism
shRNA	small hairpin RNA
SLGT-2	sodium-glucose cotransporter-2
T2DM	type 2 diabetes mellitus
TG	triglyceride
TNF-α	tumor necrosis factor-α
TZDs	thiazolidinediones
VLDL	very low-density lipoprotein
ACC	acetyl CoA carboxylase
AMP	adenosine monophosphate
ARK5	AMPK-related protein kinase 5
ASO	antisense oligonucleotide
ATP	adenosine-triphosphate
Bas	bile acids; Bcl-2, B-cell lymphoma 2
BH3	Bcl-2 homology 3
BMI	body mass index
cFLIP	Cellular FLICE (FADD-like IL-1β-converting enzyme)-inhibitory protein
COX2	cyclooxygenase-2
CVD	cardiovascular disease
DNL	*de novo* lipogenesis
ER	endoplasmic reticulum
FDA	Food and Drug Administration
FFA	free fatty acid
FMP	fecal microbiota transplantation
GLP-1	glucagon-like protein-1
HCC	hepatocellular carcinoma
HFD	high-fat diet
HMA-CoA	β-hydroxy β-methylglutaryl-CoA
IL-6	interleukin-6
IR	insulin resistance
JNK	c-Jun N-terminal kinase
LDL	low-density lipoprotein
LKB1	liver kinase B1
MAFLD	metabolic dysfunction-associated fatty liver disease
MAPK	mitogen-activated protein kinase
MUFAs	mono-unsaturated fatty acids
MSR1	macrophage scavenger receptor-1
NAFLD	non-alcoholic fatty liver disease
NASH	non-alcoholic steatohepatitis
NF-κB	nuclear factor κB
Nrf2	nuclear factor E2-related factor 2
NSAIDs	non-steroidal anti-inflammatory drugs
PPARs	peroxisome proliferator-activated receptors
PUMA	p53-upregulated modulator of apoptosis
ROS	reactive oxygen species
SCFAs	short-chain fatty acids
SNP	single-nucleotide polymorphism
shRNA	small hairpin RNA
SLGT-2	sodium-glucose cotransporter-2
T2DM	type 2 diabetes mellitus
TG	triglyceride
TNF-α	tumor necrosis factor-α
TZDs	thiazolidinediones
VLDL	very low-density lipoprotein
